# Apollo-NADP^+^ reveals in vivo adaptation of NADPH/NADP^+^ metabolism in electrically activated pancreatic β cells

**DOI:** 10.1126/sciadv.adi8317

**Published:** 2023-10-04

**Authors:** Cindy V. Bui, Curtis W. Boswell, Brian Ciruna, Jonathan V. Rocheleau

**Affiliations:** ^1^ Institute of Biomedical Engineering, University of Toronto, Toronto, Ontario, Canada.; ^2^ Toronto General Hospital Research Institute, University Health Network, Toronto, Ontario, Canada.; ^3^ Program in Developmental and Stem Cell Biology, Hospital for Sick Children, Toronto, Ontario, Canada.; ^4^ Department of Molecular Genetics, University of Toronto, Toronto, Ontario, Canada.; ^5^ Department of Genetics, Yale University School of Medicine, New Haven, CT, USA.; ^6^ Banting and Best Diabetes Centre, University of Toronto, Toronto, Ontario, Canada.

## Abstract

Several genetically encoded sensors have been developed to study live cell NADPH/NADP^+^ dynamics, but their use has been predominantly in vitro. Here, we developed an in vivo assay using the Apollo-NADP^+^ sensor and microfluidic devices to measure endogenous NADPH/NADP^+^ dynamics in the pancreatic β cells of live zebrafish embryos. Flux through the pentose phosphate pathway, the main source of NADPH in many cell types, has been reported to be low in β cells. Thus, it is unclear how these cells compensate to meet NADPH demands. Using our assay, we show that pyruvate cycling is the main source of NADP^+^ reduction in β cells, with contributions from folate cycling after acute electrical activation. INS1E β cells also showed a stress-induced increase in folate cycling and further suggested that this cycling requires both increased glycolytic intermediates and cytosolic NAD^+^. Overall, we show in vivo application of the Apollo-NADP^+^ sensor and reveal that β cells are capable of adapting NADPH/NADP^+^ redox during stress.

## Teaser

Imaging the Apollo-NADP^+^ sensor in zebrafish embryos enables in vivo measurement of endogenous β cell NADPH/NADP^+^ dynamics.

## INTRODUCTION

NADPH [reduced form of nicotinamide adenine dinucleotide phosphate (NADP^+^)] is a redox cofactor involved in many important cellular processes, including metabolism, proliferation, and antioxidant defense (*1*). Oxidized NADP^+^ is reduced back to NADPH in most cell types by the pentose phosphate pathway. Pancreatic β cells express low pentose phosphate pathway activity, which makes the NADPH/NADP^+^ redox dynamics of these cells of particular interest to study (*2*). Several groups have proposed that β cells rely on pyruvate cycling through isocitrate dehydrogenase and/or malic enzyme to maintain NADPH/NADP^+^ redox homeostasis and cell survival (*3*, *4*). Our laboratory recently showed that cytoplasmic NADP^+^ reduction also depends on mitochondrial membrane potential and NADP^+^ reduction by nicotinamide nucleotide transhydrogenase activity (*5*). During chronic stress associated with increased insulin secretion, higher levels of NADPH are needed to prevent the accumulation of reactive oxygen species, and it is unclear how β cells meet these demands to avoid dysfunction (*6*).

Despite the importance of NADPH/NADP^+^ redox homeostasis to cell survival, redox dynamics are understudied due to limitations in methodology. Several genetically encoded sensors were developed recently to address these limitations (*7*). Our group developed Apollo-NADP^+^, a genetically encoded sensor that measures NADPH/NADP^+^ redox changes as reflected in the steady-state fluorescence anisotropy due to homologous fluorescence resonance energy transfer (homo-FRET) (*8*). The design of Apollo-NADP^+^ is based on enzymatic inactivation and fluorescent protein tagging of glucose-6-phosphate dehydrogenase (G6PD), an endogenous cellular sensor for NADPH/NADP^+^ redox (*9*, *10*). Allosteric activation by NADP^+^ induces the homodimerization of Apollo-NADP^+^, resulting in homo-FRET between the homologous fluorescent protein tags. Homo-FRET depolarizes the fluorescence emission such that oxidation of NADPH can be quantified as a drop in the steady-state fluorescence anisotropy calculated from the fluorescence intensity parallel (*I*_∥_) and perpendicular (*I*_⊥_) to the excitation polarization. Anisotropy sensors effectively provide live cell ratiometric imaging with high spatial-temporal resolution and low consumption of the spectral bandwidth (i.e., single-color excitation and emission). Although Apollo-NADP^+^ previously showed some promise for monitoring NADPH/NADP^+^ redox state deep in tissues, light scattering can obscure sensor readout and presents a potential challenge to translating the use of anisotropy sensors in vivo (*8*).

Here, we translate the Apollo-NADP^+^ sensor in vivo to zebrafish embryos imaged in microfluidic devices to follow endogenous NADPH/NADP^+^ redox dynamics. Zebrafish are an ideal animal model for this work due to their genetic tractability, optical transparency for fluorescence imaging, rapid development, and genetic similarity to humans (*11*, *12*). At 5 days postfertilization (dpf), zebrafish embryos are 3.9 mm in length and have a principal pancreatic islet with morphology and function similar to mammalian pancreatic islets (*13*–*15*). The relatively small size of zebrafish at embryonic stages enables their use in microfluidic devices for stable positioning against a coverslip during imaging as well as for effective and efficient delivery of treatments. This system allowed us to take advantage of the high spatial-temporal resolution capabilities of Apollo-NADP^+^, develop an assay to characterize the NADP^+^ reduction capacity of endogenous β cells, and investigate changes in response to acute electrical activation.

## RESULTS

### Monomer and tandem-dimer fluorescent proteins can be resolved by anisotropy imaging in vivo

**Fig. 1.**
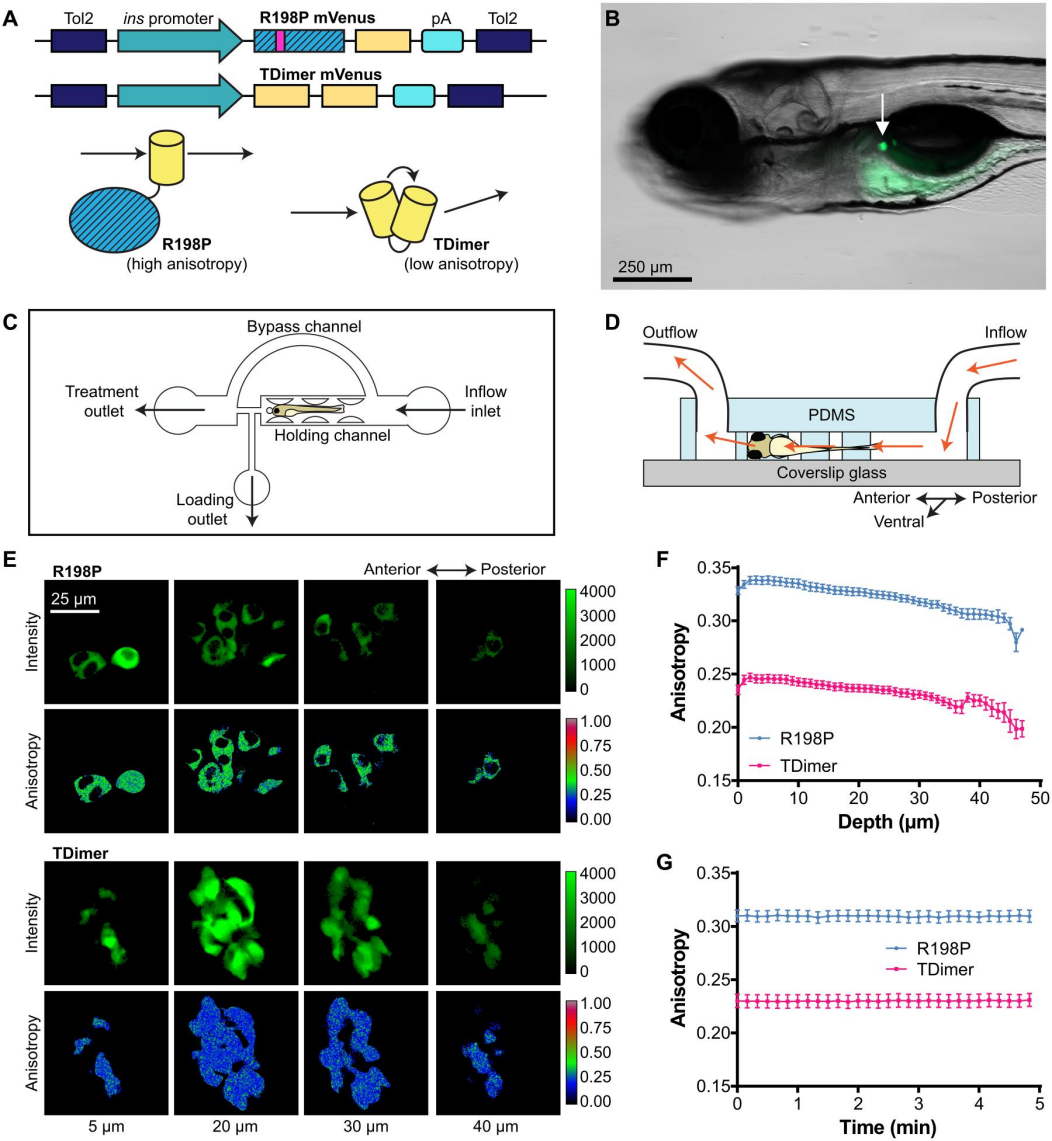
In vivo imaging of fluorescence anisotropy controls expressed in pancreatic β cells of 5-dpf zebrafish embryos. (**A**) Top: Tol2 transposon constructs used to express two fluorescence anisotropy controls, R198P and TDimer, in zebrafish. Expression is targeted to β cells using a zebrafish insulin promoter. Bottom: The R198P and TDimer establish the upper and lower bounds, respectively, of the Apollo-NADP^+^ dynamic range. pA, polyadenylation signal. (**B**) Transgenic zebrafish embryo at 5 dpf, imaged from the right lateral view. White arrow indicates the pancreatic islet. (**C**) Schematic of microfluidic device and features used to hold zebrafish embryos during imaging. (**D**) Schematic of microfluidic device cross section and perfusion flow through the device. PDMS, polydimethylsiloxane. (**E**) In vivo pancreatic islet slice images from 5-dpf R198P (top) and TDimer (bottom) transgenic zebrafish embryos, taken at various depths (5 to 40 μm). (**F**) In vivo fluorescence anisotropy imaging of 5-dpf islets of R198P and TDimer transgenic zebrafish embryos, taken at 1-μm intervals. *n* = 25 to 27 embryos. (**G**) In vivo time series fluorescence anisotropy imaging of a single islet slice from 5-dpf R198P and TDimer transgenic zebrafish embryos, taken at 10-s intervals. *n* = 13 to 15 embryos.

The Apollo-NADP^+^ sensor provides readouts of NADPH/NADP^+^ redox state through changes in steady-state fluorescence anisotropy as the sensor transitions between a monomer and homodimer state, dependent on the presence of NADP^+^ (*8*). However, steady-state fluorescence anisotropy skews downward with light scatter such as when imaging deep in tissue (*8*). To determine the impact of in vivo light scatter on the anisotropy-based Apollo-NADP^+^ sensor, we imaged the steady-state fluorescence anisotropy of cytosolic monomeric and tandem-dimeric mVenus fluorescent protein constructs expressed in pancreatic β cells of living zebrafish embryos (Fig. 1). Transgenic lines with zebrafish insulin promoter–driven β cell expression of a monomeric mutant of mVenus-tagged Apollo-NADP^+^ [Tg(*ins*:Apollo-NADP^+^-R198P), abbreviated as R198P] and a tandem dimer of mVenus fluorescent protein [Tg(*ins*:TDimer), abbreviated as TDimer] were generated using Tol2-mediated transgenesis (Fig. 1A) (*16*, *17*), and stable generations of each reporter were imaged at the 5-dpf stage. The R198P mutation renders the sensor unresponsive to NADP^+^ (*8*, *18*), causing the sensor to stay in the monomeric state (showing high fluorescence anisotropy), while TDimer encodes two mVenus fluorescent proteins linked in series to form pairs of fluorophores that homo-FRET (showing low fluorescence anisotropy). Thus, the R198P and TDimer reporters act as monomer and homodimer anisotropy controls and establish the upper and lower bounds, respectively, of the dynamic range of Apollo-NADP^+^ steady-state fluorescence anisotropy. The principal islet of 5-dpf R198P and TDimer transgenic zebrafish embryos can be identified under fluorescence (Fig. 1B). The embryos were positioned by a microfluidic device against a coverslip to stabilize the embryos at a consistent working distance during imaging (Fig. 1, C and D). Two-photon imaging of R198P and TDimer in live embryos shows that the upper and lower range of the sensor can be clearly distinguished by anisotropy while imaging the entire islet (Fig. 1, E and F). The two copies of mVenus per TDimer also resulted in greater fluorescence intensity observed in islets of TDimer embryos compared to the islets of R198P embryos (fig. S1, A and B). Data collected from multiple transgenic founder lines were compared to confirm that the difference in fluorescence intensity was not due to transgene position (fig. S1A). The anisotropies of both constructs were relatively stable over most of the islet, with some downward skewing occurring at the extremities, showing that light scatter and depth affect the absolute anisotropy but have limited effect on the dynamic range (Fig. 1F). The anisotropies of both constructs were particularly stable when imaging a single slice over time (Fig. 1G). The average intensities of these constructs vary when imaging over depth (fig. S1A) but are relatively stable when imaging over time, consistent with limited two-photon photobleaching (fig. S1B). These data show that the dynamic range of anisotropy sensors is maintained in vivo over the depth of an islet and that these sensors are particularly amenable to imaging over time at a single depth of β cells in 5-dpf zebrafish embryos.

### Apollo-NADP^+^ can be used to measure endogenous β cell NADPH/NADP^+^ dynamics

**Fig. 2.**
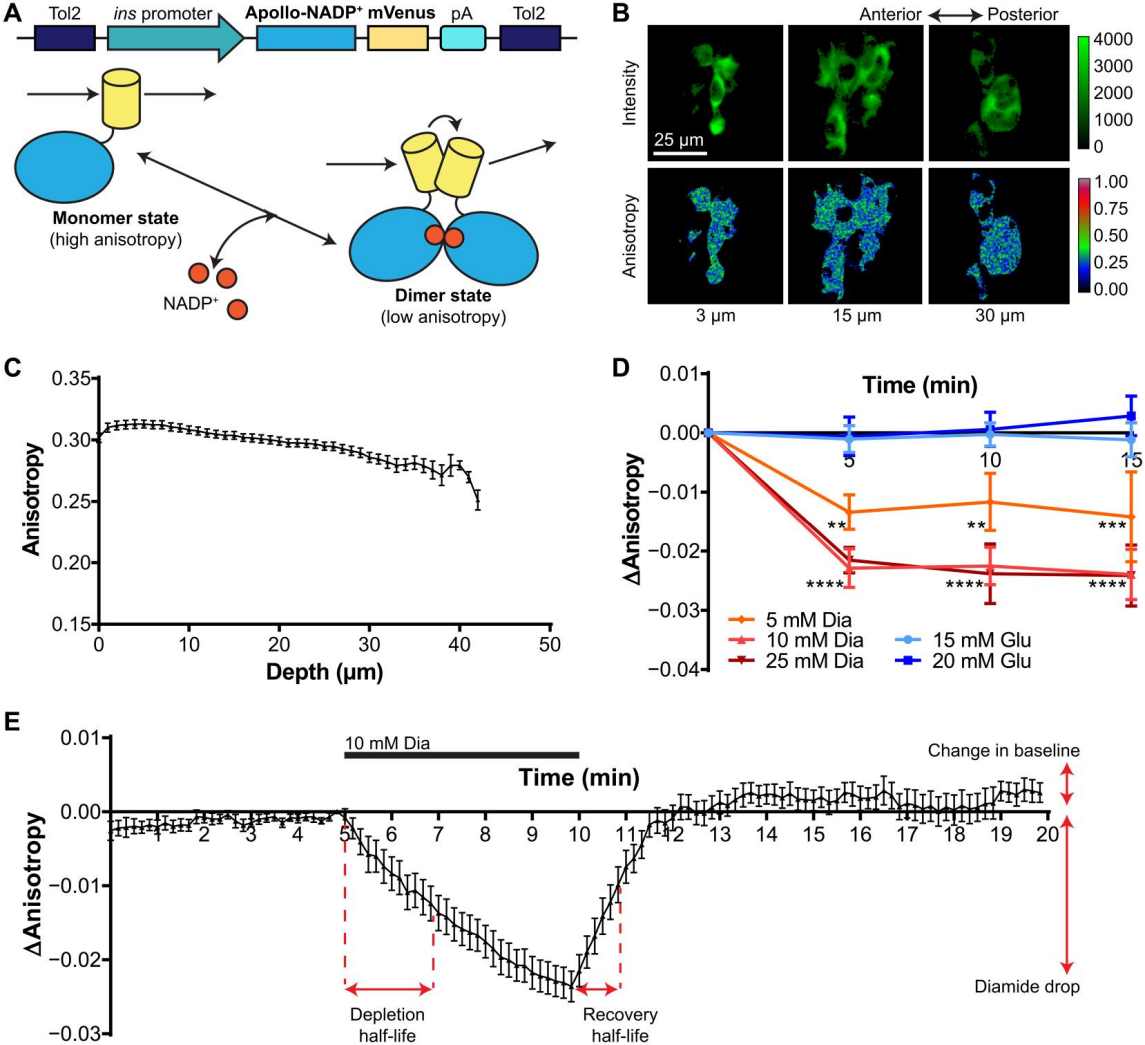
In vivo imaging of mVenus-tagged Apollo-NADP^+^ expressed in pancreatic β cells of 5-dpf zebrafish embryos. (**A**) Top: Tol2 transposon construct used to express Apollo-NADP^+^ in zebrafish. Expression is targeted to β cells using a zebrafish insulin promoter. Bottom: Apollo-NADP^+^ switches between a monomer and homodimeric state in response to changes in NADP^+^ concentration. (**B**) In vivo pancreatic islet slice images from 5-dpf Apollo-NADP^+^ transgenic zebrafish embryos, taken at 3-, 15-, and 30-μm depths. (**C**) In vivo fluorescence anisotropy imaging of 5-dpf islets of Apollo-NADP^+^ zebrafish embryos, taken at 1-μm intervals. *n* = 26 embryos. (**D**) In vivo time series fluorescence anisotropy imaging of 5-dpf islets of Apollo-NADP^+^ transgenic zebrafish in response to treatment with glucose (Glu) or diamide (Dia), taken at 5-min intervals. *n* = 8 to 15 embryos; **P < 0.01 and ****P < 0.0001. (**E**) In vivo time series fluorescence anisotropy imaging of a single islet slice from 5-dpf Apollo-NADP^+^ transgenic zebrafish embryos, taken at 10-s intervals. Diamide (10 mM) was added at 5 min and removed at 10 min. *n* = 15 embryos.

We next wanted to confirm that the Apollo-NADP^+^ sensor itself would be functional in vivo (Fig. 2). Transgenic zebrafish with insulin promoter–driven β cell expression of mVenus-tagged Apollo-NADP^+^ [Tg(*ins*:Apollo-NADP^+^)] were generated using Tol2-mediated transgenesis, and stable generations were imaged at 5 dpf (Fig. 2A, top). Apollo-NADP^+^ switches between monomeric and homodimeric states in response to changes in free NADP^+^ concentration, such that the fluorescence anisotropy is high when the NADPH/NADP^+^ ratio is high and the fluorescence anisotropy is low when the NADPH/NADP^+^ ratio is low (Fig. 2A, bottom). Imaging cytosolic Apollo-NADP^+^ at various heights within the islet shows similar expression patterns and intensities to the R198P (Fig. 2B and fig. S1A). This is expected due to the equal sequence length and presence of a single copy of mVenus on both constructs. Two-photon anisotropy imaging of Apollo-NADP^+^ in β cells of live 5-dpf embryos shows a similar anisotropy over much of the islet, with some downward skewing at larger depths (Fig. 2, B and C). The relatively high anisotropy of Apollo-NADP^+^ suggested elevated β cell NADPH levels, likely due to normal blood glucose and nutrient supply from the embryonic gut at 5 dpf, and this is consistent with no external feeding being required at this stage of embryonic development (*19*). To validate the high NADPH basal state of Apollo-NADP^+^ and that the sensor could track changes in NADPH/NADP^+^, we perfused 5-dpf embryos in the microfluidic device with glucose and diamide to reduce and oxidize β cell NADPH/NADP^+^ state, respectively (Fig. 2D). These data show no change in response to glucose but a rapid diamide-stimulated drop in anisotropy consistent with NADPH oxidation that plateaued within 5 min (Fig. 2D). To determine whether the sensor could also track reduction events in vivo, we treated embryos with *N*-acetyl-l-cysteine ethyl ester (NACET) before diamide oxidation (fig. S2). NACET is a derivative of the antioxidant drug, *N*-acetyl-l-cysteine with improved pharmacological kinetics and has previously been used in zebrafish studies (*20*, *21*). These data show that the diamide-stimulated drop in anisotropy was blocked with 1 mM NACET and partially blocked at lower concentrations, demonstrating the ability of Apollo-NADP^+^ to distinguish between a gradient of redox states. To explore the underlying metabolism supplying the NADPH/NADP^+^ redox state, we transiently treated the zebrafish embryos with 10 mM diamide to measure the oxidation and reduction dynamics (Fig. 2E). The embryos were perfused with egg water for 5 min to establish a stable baseline β cell anisotropy followed by transient (5 min) diamide-induced oxidation and 10 min of recovery in egg water. These data show the average trace from 15 embryos, highlighting the precision and consistency of anisotropy sensing while also revealing the recovery half-life dynamics and return to baseline due to normal underlying metabolism. In particular, recovery after the removal of diamide resulted in the NADPH/NADP^+^ redox state returning to equilibrium within 5 min (Fig. 2E). As expected, the R198P and TDimer embryos constructs were unaffected by this NADPH oxidation assay, showing no changes in anisotropy with diamide treatment and demonstrating the specificity of Apollo-NADP^+^ in reporting changes to NADPH/NADP^+^ redox state (fig. S1C). We postulate that these Apollo-NADP^+^ traces reflect the underlying metabolism supporting NADPH/NADP^+^ redox homeostasis. These data validate the Apollo-NADP^+^ sensor in zebrafish pancreatic β cells while showcasing an assay using diamide-induced oxidation to track real-time NADPH recovery.

### Pyruvate cycling is the primary NADP^+^ reduction pathway in 5-dpf zebrafish β cells

**Fig. 3.**
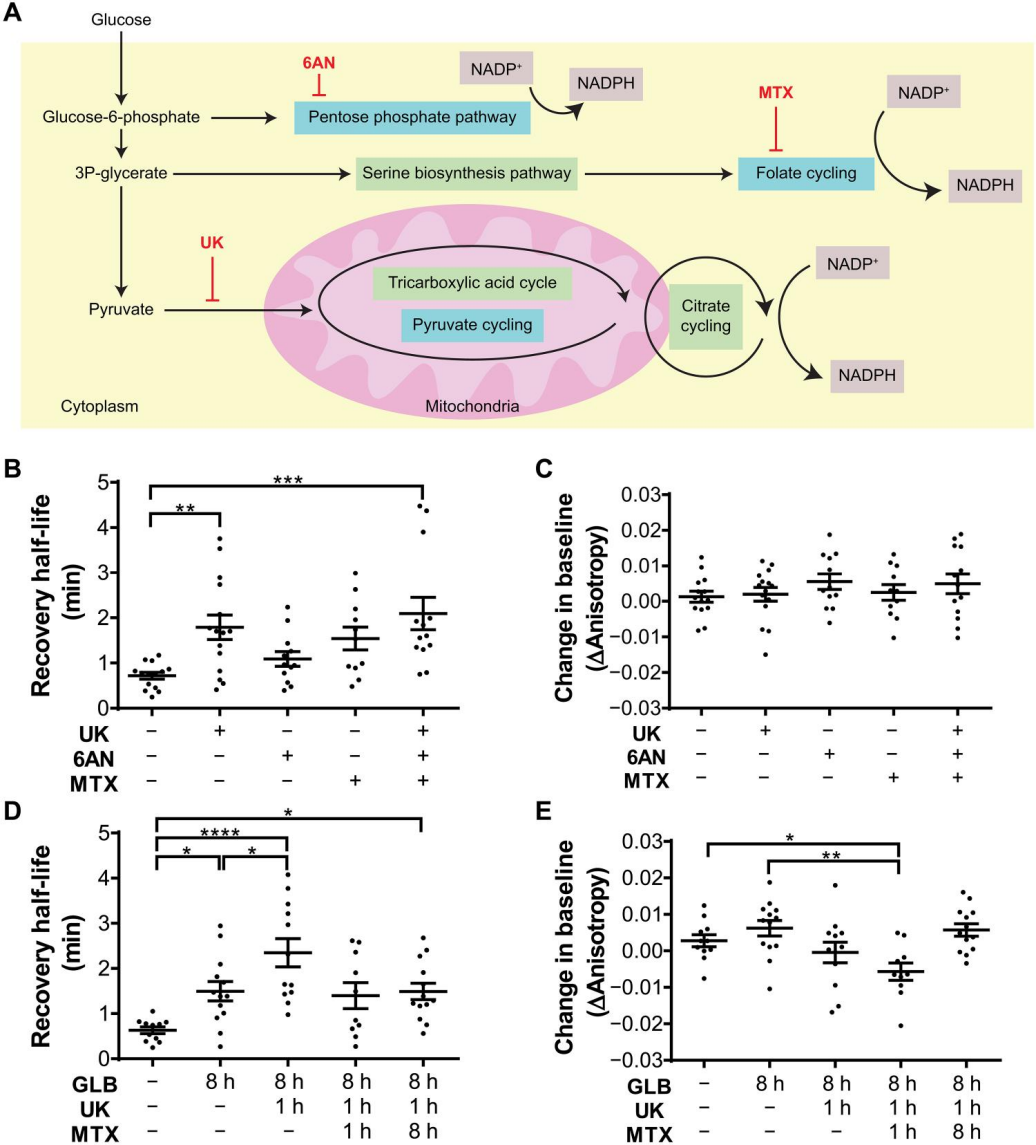
Selective inhibition of NADP^+^ reduction pathways in unstressed and stressed pancreatic β cells of 5-dpf zebrafish embryos. (**A**) Diagram of NADP^+^ reduction pathways, indicated with blue shading, and associated chemical inhibitors, indicated with red text. Connected pathways are indicated with green shading, and mitochondria are indicated with pink shading. (**B**) NADPH recovery half-life after diamide removal, quantified from in vivo time series fluorescence anisotropy imaging of islet slices from 5-dpf Apollo-NADP^+^ transgenic zebrafish embryos. UK (200 μM) was used to inhibit pyruvate cycling, 6AN (50 μM) was used to inhibit pentose phosphate pathway, and MTX (50 μM) was used to inhibit folate cycling. Chemical inhibitors were added 1 hour before imaging. (**C**) Change in baseline anisotropy, quantified from in vivo time series fluorescence anisotropy imaging of islet slices from 5-dpf Apollo-NADP^+^ transgenic zebrafish embryos. (**D**) NADPH recovery half-life after diamide removal, quantified from in vivo time series fluorescence anisotropy imaging of islet slices from 5-dpf Apollo-NADP^+^ transgenic zebrafish embryos. Stress was induced by 8 hours of treatment with 20 μM GLB. (**E**) Change in baseline anisotropy, quantified from in vivo time series fluorescence anisotropy imaging of islet slices from 5-dpf Apollo-NADP^+^ transgenic zebrafish embryos. *n* = 10 to 15 embryos. *P < 0.05, **P < 0.01, ***P < 0.001, and ****P < 0.0001.

In pancreatic β cells, low pentose phosphate pathway activity would leave pyruvate cycling to be the main source of NADP^+^ reduction (*2*–*4*). To investigate the contribution of individual pathways to NADP^+^ reduction, we performed diamide-induced oxidation assays in 5-dpf Apollo-NADP^+^ zebrafish embryos with selective inhibition of potential pathways of NADP^+^ reduction (Fig. 3). Chemical inhibitors were used to inhibit the pentose phosphate pathway, pyruvate cycling, and folate cycling (Fig. 3A). 6-Aminonicotinamide (6AN) was used to preferentially target the 6-phosphogluconate dehydrogenase enzyme and inhibit pentose phosphate pathway activity (*22*–*25*). UK5099 (UK) was used to inhibit the mitochondrial pyruvate carrier, blocking pyruvate entry into the mitochondria and disrupting pyruvate cycling activity mediated by both malic and citrate enzymes (*26*, *27*). Methotrexate (MTX) was used to inhibit dihydrofolate reductase, a critical folate cycling enzyme (*28*–*31*). As per the assay, 5 min of baseline anisotropy measurements were taken, followed by depleting embryos of NADPH by 5 min of diamide-induced oxidation and 10 min of recovery. The relative drop in anisotropy due to oxidation by diamide and the kinetics of diamide-stimulated NADPH depletion (i.e., temporal half-life) were not significantly different between treatment groups, consistent with first-order oxidation of NADPH being solely dependent on diamide concentration (fig. S3, A and B). In contrast, quantifying the recovery time half-life (based on the anisotropy between the 10- and 20-min time points) showed that only the UK-treated embryos had significantly slower recovery time (Fig. 3B). Embryos treated with either 6AN or MTX showed slight impairments in recovery speed, and embryos treated with all three inhibitors showed greater impairment than inhibition of pyruvate cycling using UK alone. There were no significant differences between treatment groups when comparing the change in baseline anisotropy at 20 min to the baseline at 5 min (Fig. 3C). These data suggest that pyruvate cycling is the primary source of NADP^+^ reduction in β cells in vivo; however, these data also suggest that the pentose phosphate pathway and folate cycling may be active to a lesser extent under these normal conditions.

### Acute electrical stress activates additional NADP^+^ reduction in 5-dpf zebrafish β cells

During stress, the demand for NADPH is elevated, and yet it is unclear how β cell metabolism meets this demand to avoid dysfunction (*6*). Other groups have shown that activation of cytosolic folate cycling is sensitive to a variety of substrate availability changes, and we hypothesized that substrate changes during stress may similarly induce activation of cytosolic folate cycling in β cells for additional NADP^+^ reduction capacity (*32*–*35*). To mimic β cell activation and induce a stressed state, we treated 5-dpf Apollo-NADP^+^ zebrafish embryos with ATP-sensitive potassium channel (K_ATP_) inactivator, glibenclamide (GLB), for 8 hours, followed by performing diamide-induced oxidation assays (Fig. 3, D and E). We compared NADPH/NADP^+^ dynamics of GLB-treated embryos with and without GLB present during assay imaging to confirm that there were no significant effects on the dynamics due to endogenous blood glucose regulation in vivo (fig. S4). These data again show no difference in the diamide-stimulated relative drop in anisotropy and depletion half-life between the different treatment groups, consistent with diamide concentration being the dominant factor in the depletion dynamics (fig. S3, C and D). In contrast, GLB-treated embryos showed a slower recovery rate (i.e., higher recovery half-life) of NADP^+^ reduction (Fig. 3D). This recovery was slowed further by inhibition of pyruvate cycling using UK, indicating that pyruvate cycling is still a major route of NADP^+^ reduction in stressed β cells. In contrast, inhibition of folate cycling using MTX showed no change in the recovery rate but a significant drop in the final recovery equilibrium (Fig. 3E). These data suggest that 8 hours of electrical activation triggers folate cycling to raise the final NADPH/NADP^+^ state rather than increasing the NADP^+^ reduction rate. Recent studies with chronic MTX pretreatment in mice demonstrated that MTX was capable of inhibiting nicotinamide adenine dinucleotide kinase (NADK), an enzyme that catalyzes phosphorylation of nicotinamide adenine dinucleotide (oxidized form) (NAD^+^) to NADP^+^ (*36*–*38*). To determine whether the NADK-induced expansion of NADP^+^ pools could contribute to the change in baseline, we treated embryos with both GLB and MTX for 8 hours (Fig. 3, D and E). This chronic treatment with MTX blocked the change in baseline observed in embryos with acute MTX treatment, suggesting that NADK activation during stress expands the pool of NADP^+^ in β cells. Overall, these data suggest that β cells respond to 8 hours of stress with a combination of activity from NADK and folate cycling.

### Apollo-NADP^+^ responses in 5-dpf zebrafish embryonic β cells are consistent with responses in INS1E β cells

**Fig. 4.**
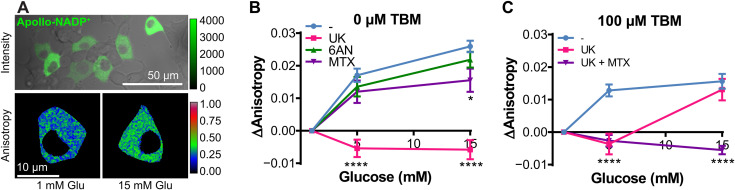
Selective inhibition of NADP^+^ reduction pathways in unstressed and stressed INS1E pancreatic β cells. (**A**) INS1E β cells transfected with Apollo-NADP^+^. Top: Fluorescence intensity image of Apollo-NADP^+^ expressed in INS1E β cells. Bottom: Fluorescence anisotropy images of Apollo-NADP^+^ expressing INS1E β cells stimulated with glucose. (**B**) Glucose-stimulated NADP^+^ reduction of INS1E β cells transfected with Apollo-NADP^+^. UK (50 μM) was used to inhibit pyruvate cycling, 6AN (5 μM) was used to inhibit the pentose phosphate pathway, and MTX (5 μM) was used to inhibit folate cycling. Chemical inhibitors were added 1 hour before imaging. (**C**) Glucose-stimulated NADP^+^ reduction of INS1E β cells transfected with Apollo-NADP^+^. Stress was induced by 8 hours of stress treatment with 100 μM TBM. *n* = 3 to 6 replicates. *P < 0.05 and ****P < 0.0001.

To validate our zebrafish findings, we investigated NADP^+^ reduction pathways in rat insulinoma (INS1E) β cells transfected with mVenus-tagged Apollo-NADP^+^ (Fig. 4). After 1 hour of starvation in 1 mM glucose, Apollo-NADP^+^ expressed in the cytosol of INS1E β cells showed low anisotropy consistent with a substrate-limited redox state. The cells showed a rise in anisotropy upon stimulation with 15 mM glucose (Fig. 4A). Comparing the glucose-stimulated response in the presence of metabolic inhibitors, only UK treatment blocked the glucose-stimulated NADP^+^ reduction, consistent with pyruvate cycling being the primary contributor to β cell NADP^+^ reduction (Fig. 4B). Cells treated with 6AN or MTX showed minor decreases in the glucose-stimulated reduction of NADP^+^, consistent with low pentose phosphate pathway and folate cycling activity. These data agree with our data from the β cells of 5-dpf zebrafish (Fig. 3B). These data were further validated by performing glucose stimulation in Apollo-NADP^+^–transfected INS1E β cells treated with alternative inhibitors (fig. S5A). 7-Aminocarboxycoumarin 2 (7ACC2) is an inhibitor of mitochondrial pyruvate carrier and was used as an alternative inhibitor of mitochondrial pyruvate transport and pyruvate cycling (*27*, *39*). LY345899 (LY) is an inhibitor of methylenetetrahydrofolate dehydrogenase 1, a cytosolic NADP^+^-reducing enzyme in the folate cycle (*40*–*42*). As expected, these data show that glucose-stimulated NADP^+^ reduction was blocked with 7ACC2-induced inhibition of pyruvate cycling and not blocked with LY-induced inhibition of folate cycling. To induce a stressed state through β cell activation, we treated INS1E β cells with a K_ATP_ channel inactivator, tolbutamide (TBM), for 8 hours (Fig. 4C). In TBM-stressed INS1E β cells, inhibition of pyruvate cycling with UK significantly blocked NADP^+^ reduction at low glucose (5 mM glucose) but did not block NADP^+^ reduction at high glucose (15 mM). These data are consistent with NADP^+^ reduction at high-glucose stimulation by alternative pathways. Inhibition of folate cycling with the addition of MTX shows a blocked response across glucose concentrations. These data are consistent with 8 hours of stress triggering increased folate activity in β cells, similar to what was observed in GLB-treated 5-dpf zebrafish embryos (Fig. 3E). These data were further validated by replacing MTX with LY (fig. S5B). As expected, these data show that addition of LY-induced inhibition of folate cycling blocked high-glucose–stimulated NADP^+^ reduction in stressed cells.

### Additional NADP^+^ reduction during stress is enabled by a combination of both glycolytic intermediates and NAD^+^ supply

**Fig. 5.**
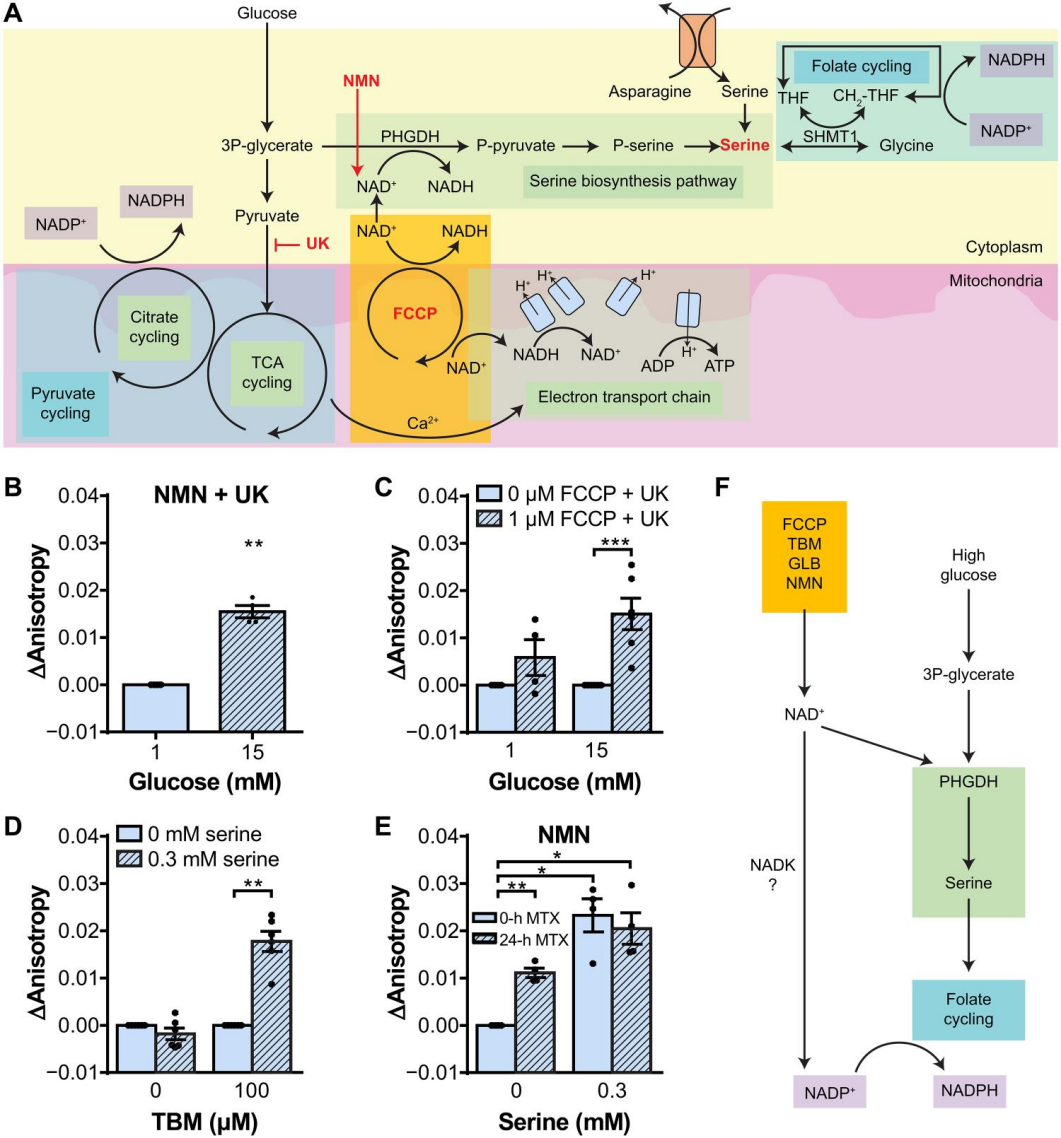
Investigating potential mechanisms of folate cycle activation during chronic stress in INS1E β cells. (**A**) Diagram showing sources of NAD^+^ and serine for folate cycling activation. ADP, adenosine 5′-diphosphate; ATP, adenosine 5′-triphosphate; TCA, tricarboxylic acid; SHMT1, serine hydroxymethyltransferase 1; CH_2_-THF, 5,10-methylenetetrahydrofolate; P-pyruvate, 3-phosphohydroxypyruvate; P-serine, 3-phosphoserine. (**B**) Glucose stimulation of INS1E β cells transfected with Apollo-NADP^+^. Cells were treated with 100 μM NMN for 24 hours to elevate NAD^+^ levels. UK (50 μM) was added 1 hour before imaging to block NADP^+^ reduction by pyruvate cycling. (**C**) Carbonyl cyanide *p*-trifluoromethoxyphenylhydrazone (FCCP; 1 μM) stimulation of INS1E β cells transfected with Apollo-NADP^+^ at 1 or 15 mM glucose. UK (50 μM) was added 1 hour before imaging to block pyruvate cycling. (**D**) Serine stimulation (0.3 mM) of unstressed (0 μM TBM) and stressed (8 hours of 100 μM TBM) INS1E β cells transfected with Apollo-NADP^+^. Cells were pretreated with 1 mM asparagine for 1 hour before imaging to promote extracellular serine uptake. Imaging was done at low glucose (1 mM) to avoid activation of pyruvate cycling. (**E**) Serine stimulation (0.3 mM) of INS1E β cells transfected with Apollo-NADP^+^. Cells were treated with 100 μM NMN for 24 hours to elevate NAD^+^ levels or treated with both 100 μM NMN and 5 μM MTX for 24 hours to block NAD^+^ conversion to NADP^+^. One hour before imaging, cells were pretreated with 1 mM asparagine to promote extracellular serine uptake. Imaging was done at low glucose (1 mM) to avoid activation of pyruvate cycling. (**F**) Simplified diagram of potential factors required to trigger NADP^+^ reduction through folate cycling. *n* = 3 to 6 replicates. *P < 0.05, **P < 0.01, and ***P < 0.001.

Folate cycling reduces cytoplasmic NADP^+^ when supplied with serine (*32*). Thus, a potential mechanism for glucose-stimulated folate cycling during metabolic stress is through activation of the serine biosynthesis pathway (Fig. 5A). The serine biosynthesis pathway bridges glycolysis and folate cycling, with activation of phosphoglycerate dehydrogenase (PHGDH) as the first and rate-limiting step (*43*, *44*). PHGDH activity primarily depends on the availability of NAD^+^ and glycolytic intermediate 3P-glycerate (3PG) (*43*, *44*). To determine whether NAD^+^ availability limits folate cycling in unstressed β cells, we treated INS1E β cells with NAD^+^ precursor, β-nicotinamide mononucleotide (NMN), for 24 hours to elevate NAD^+^ levels (Fig. 5B). We subsequently measured the glucose-simulated response in the presence of UK to both elevate 3PG and block pyruvate cycling-induced NADP^+^ reduction. These data show NADP^+^ reduction in the absence of pyruvate cycling, suggesting that increasing the supply of both NAD^+^ and 3PG activated PHGDH and folate cycling.

Prolonged β cell activation and increased glucose metabolism can lead to mitochondrial electron transport chain (ETC) uncoupling, elevating cytosolic NAD^+^ and increasing availability of the glycolytic intermediate 3PG to activate PHGDH for serine biosynthesis. To determine whether ETC uncoupling could trigger NADP^+^ reduction external to pyruvate cycling, we treated INS1E β cells with 1 μM carbonyl cyanide *p*-trifluoromethoxyphenylhydrazone to partially uncouple the mitochondria and UK to exclude pyruvate cycling contributions from the measurements (Fig. 5C). These data show glucose-stimulated NADP^+^ reduction in the absence of pyruvate cycling with mild ETC uncoupling.

Next, we wanted to determine whether serine, the downstream product of PHGDH activation, could stimulate NADP^+^ reduction outside of pyruvate cycling. We pretreated β cells with asparagine to facilitate subsequent extracellular serine uptake through membrane transporter amino acid exchange (Fig. 5D) (*45*). These data show that serine alone was not sufficient to induce NADP^+^ reduction unless the cells were prestressed with TBM treatment. This suggests that NAD^+^ and/or 3PG buildup may have a secondary role in order for serine to trigger NADP^+^ reduction. We similarly measured INS1E β cells transfected with mitochondrial-localized Apollo-NADP^+^ to determine whether extracellular serine is transported to the mitochondria under normal conditions and diverted during stress (fig. S5). These data show that, while serine induces mitochondrial NADPH oxidation, TBM treatment does not cause a change in mitochondria NADPH/NADP^+^ dynamics.

Our data from zebrafish embryos suggested contribution from NADK activity during stress (Fig. 3, D and E). NADK is sensitive to NAD^+^ concentration (*36*–*38*), so to determine whether NAD^+^ may have a dual role of activating both serine biosynthesis and NADK, we treated INS1E β cells with NMN or a combination of NMN and MTX before stimulation with extracellular serine (Fig. 5E). These cells were again pretreated with asparagine to facilitate serine uptake and imaged at low glucose to avoid activation of pyruvate cycling. These data show that the elevation of NAD^+^ by NMN enabled serine-stimulated NADP^+^ reduction. Cells treated with both NMN and MTX showed a more reduced NADPH/NADP^+^ state before serine stimulation and blunted serine-stimulated NADP^+^ reduction. This suggests that, while NAD^+^-induced activation of NADK causes the NADPH/NADP^+^ state to be more oxidized, the effects are neutralized by NAD^+^ and serine supply for folate cycling. The basal anisotropy was compared between these treatment groups, and untreated cells to confirm asparagine treatment did not affect interpretations of the baseline NADPH/NADP^+^ state between these treatment groups (fig. S7). Overall, these data suggest that NAD^+^ generated during stress may target both PHGDH for additional NADP^+^ reduction through folate cycling and NADK for NADP^+^ generation as a reduction substrate (Fig. 5F).

## DISCUSSION

In this study, we expressed the Apollo-NADP^+^ sensor in live zebrafish embryos and developed a diamide-oxidation assay to measure endogenous NADPH/NADP^+^ redox dynamics. This assay enabled us to compare β cell NADP^+^ reduction under inhibition of individual metabolic pathways following NADPH oxidation by diamide. The data support the notion that cytosolic NADP^+^ reduction in β cells is primarily through pyruvate cycling rather than the pentose phosphate pathway. We show that, after 8 hours of electrical stress, there is an increase in cytosolic folate cycling to increase NADPH/NADP^+^ reduction.

Although many genetically encoded sensors are available for live cell imaging, their use has largely been limited to in vitro applications. This limitation is likely due to difficulties in controlling in vivo sensor expression and concerns regarding light scattering in tissues (*8*, *46*, *47*). Light scattering and low expression can obfuscate sensor measurements by decreasing the signal-to-noise ratio. On the other end of the spectrum, substrate sequestering by hyperexpression of the sensor can interfere with endogenous metabolism. Apollo-NADP^+^ is a fluorescence anisotropy–based sensor with a ratiometric readout and, therefore, is less affected by variations in expression compared to fluorescence intensity–based sensors but is sensitive to light scatter (*8*). We showed that we could discern changes in cytosolic NADPH/NADP^+^ redox dynamics using Apollo-NADP^+^ expressed in genetically tractable and optically transparent zebrafish embryos and that β cell–specific measurements could be made using a zebrafish insulin promoter (*16*). We expect that the use of the sensor could be extended to other cell types by replacing the insulin promoter with alternative promoter sequences. Furthermore, our laboratory recently showed that the sensor may be targeted to subcellular organelles via the addition of organelle localization sequences (*5*). With the use of a microfluidic device, we were able to reduce noise in the measurements by stabilizing the animals in a microfluidic device against a coverslip during imaging and treatment delivery. With this combination of techniques, we developed an in vivo assay where transient treatment with and recovery from diamide is used to perturb and track the real-time dynamics of NADPH oxidation and NADP^+^ reduction. The ability to use a genetically encoded fluorescence sensor in vivo enables the observation of live cellular events in real time, revealing insights and nuances that would not be captured in vitro. This is a substantial advantage over endpoint in vitro biochemical assays that rely on fixed cell samples.

Using our in vivo diamide-oxidation assay, we isolated the contribution of several metabolic pathways to NADP^+^ reduction and showed that the majority of β cell NADP^+^ reduction is through pyruvate cycling. We revealed that 8 hours of electrical activation induced additional NADP^+^ reduction through an increase in folate cycling activity at high glucose. These findings were further explored in INS1E cells where we confirmed that folate cycling requires both elevated glucose metabolism and elevated cytosolic NAD^+^. These factors are required for the activation of PHGDH and serine biosynthesis, upstream of folate cycling (*43*, *44*). The shift in NADPH/NADP^+^ equilibrium demonstrated by stressed β cells in vivo suggested that the cytosolic pools of available NADPH/NADP^+^ were altered by stress. We identified NADK as a likely cause for this phenomenon, due to its sensitivity to NAD^+^ concentration and ability to convert NAD^+^ to NADP^+^ (*36*–*38*). Our data suggest that elevated NAD^+^ levels during stress activate both serine production and NADK. This was explored through chronic and acute application of MTX to block NADP^+^ accumulation by NADK over the course of a stress treatment (8 hours) and to block NADP^+^ reduction by folate cycling during glucose stimulation and recovery from diamide (1 hour). Our data show that elevated glucose metabolism and elevated cytosolic NAD^+^ during stress trigger serine production to enable folate cycling as an additional NADP^+^ reduction pathway and NADK activity to phosphorylate NAD^+^, expanding the pool of NADP^+^ that can be reduced.

Our data suggest that folate cycling is a potential metabolic source of NADP^+^ reduction in stressed pancreatic β cells. This adaptation could support the health and survival of these cells to prevent cellular dysfunction and diabetes progression. Genetically encoded sensors such as Apollo-NADP^+^ are powerful tools for exploring endogenous metabolic changes in living cells. Here, we demonstrated the utility of the homo-FRET sensor Apollo-NADP^+^ sensor in vivo. While it has always been theoretically possible to use these sensors in vivo, the challenges to do so have limited this application. We have shown that this can be done practically by combining the use of zebrafish embryos and microfluidics. We anticipate that the translation of other genetically encoded sensors and the development of additional in vivo assays and image processing pipelines will provide more insight into endogenous metabolism.

### Limitations

Our interpretations are primarily based on chemical inhibition experiments and findings should be further validated by repeating experiments on animals where pathway activity is inhibited through enzymatic mutation, transcript silencing, or the development of a disease model. Experiments were performed in 5-dpf zebrafish embryos and in the INS1E β cell line and could be extended to mice using pancreatic imaging windows (*48*–*51*) or extended to human tissue samples through viral transduction of Apollo-NADP^+^. When using the sensor in other tissues, TDimer and R198P constructs can be used to check the dynamic range of the sensor in the environment of interest. The dynamic range of the sensor may be reduced by high levels of endogenous G6PD, in which case the anisotropy may be normalized by the dynamic range as *r*_norm_ = *r*_Apollo_/(*r*_R198P_ − *r*_TDimer_). In cases where it is difficult to distinguish between a change in NADPH/NADP^+^ ratio and/or change in NADP(H) pools, it may be necessary to perform multiparametric imaging with additional sensors within the same cells or perform endpoint biochemical assays. At 5 dpf, there is only a single islet present in the zebrafish embryo, and the heterogeneity of these β cells remains to be explored. It may be necessary to adapt machine learning algorithms to rapidly segment individual β cells at later stages in development where there are a greater number of cells and islets.

## MATERIALS AND METHODS

### Experimental design

We aimed to determine whether fluorescence anisotropy sensors could be used in vivo to explore β cell NADPH/NADP^+^ dynamics. To accomplish this aim, we generated transgenic zebrafish lines with β cell expression of Apollo-NADP^+^ and performed fluorescence anisotropy imaging in microfluidic devices. We developed an assay to measure the metabolic reduction of NADP^+^ in β cells by imaging recovery of NADPH/NADP^+^ redox state after diamide oxidation in 5-dpf zebrafish embryos. These data were subsequently compared to responses in INS1E β cells transfected with Apollo-NADP^+^.

### Zebrafish care and use in microfluidic devices

Zebrafish husbandry and experimental protocols were approved by the Hospital for Sick Children’s Animal Care Committee, and all protocols were performed in accordance with the Canadian Council on Animal Care guidelines. Zebrafish embryos were cultured at 28°C in egg water, composed of methylene blue (0.5 mg/liter) in system water. For imaging, 5-dpf zebrafish embryos were anesthetized in a dilute solution of tricaine (150 mg/liter) and loaded individually into a microfluidic device (Neofluidics). Chemical treatments were added to the egg water used to incubate embryos and perfuse them in the microfluidic device. Diamide delivery and removal from the device was done using a syringe pump with a flow rate set to 3 ml/hour. Fluorescein (50 μM) was added to the diamide solution to confirm the switching of treatments in the device using fluorescence.

### Cloning and zebrafish transgenesis

Tol2 transposon transgenesis constructs were generated using standard Tol2 kit Gateway–compatible vectors (*17*). Previously generated sequences for Apollo-NADP^+^ (*8*), TDimer (*8*), R198P (*8*), and the zebrafish insulin promoter (*16*) were cloned into entry plasmids by BP recombination. The entry plasmids containing the zebrafish insulin promoter, Apollo-NADP^+^, and an SV40 late polyadenylation signal (*52*) were subsequently shuttled into a pDEST Tol2 expression vector via LR recombination. Separate expression vectors for TDimer and R198P were generated by replacing the Apollo-NADP^+^ fragment with fragments encoding either TDimer or R198P, using the InFusion Cloning Kit. Plasmids may be requested at www.addgene.org/Jonathan_Rocheleau/. Wild-type AB and Tübingen (Tu) embryos were injected at the one-cell stage with 25 pg of plasmid and 25 pg of Tol2 transposase RNA and screened at 5 dpf for transgenesis marker expression on an AxioZoom V16 (Zeiss). Embryos showing strong fluorescence were sorted and grown to adulthood, and individuals were crossed to wild-type strains to generate separate independent stable F1 lines for each of Apollo-NADP^+^, TDimer, and R198P.

### INS1E β cell culture, transfection, and treatments

INS1E β cells were cultured in RPMI-1640 medium supplemented with 5% fetal bovine serum, 1 mM sodium pyruvate, 10 mM Hepes, penicillin (100 U/ml), streptomycin (100 μg/ml), and 50 μM β-mecaptoethanol. For imaging, cells were plated on glass-bottom dishes (MatTek) and transfected with Apollo-NADP^+^ plasmid using Lipofectamine 3000 following the manufacturer’s protocol. One hour before imaging, culture medium was replaced with the Live Cell Imaging Solution (Invitrogen) supplemented with 1 mM glucose and 0.1% bovine serum albumin. Chemical treatments were added to culture medium and imaging buffer at the indicated time points.

### Imaging

Wide-field fluorescence imaging of 5-dpf zebrafish embryos was performed on an Axio Zoom V16 (Zeiss). Two-photon fluorescence anisotropy imaging was performed on an LSM710 microscope (Zeiss) as previously described (*7*). Cell lines were imaged using a 63×/1.4 numerical aperture (NA) oil immersion objective, and zebrafish pancreatic islets were imaged using a 40×/1.3 NA oil immersion objective. For imaging of whole islets from zebrafish, a *z* stack of the islet was captured by imaging at 1-μm intervals over the depth of the islet. For time series imaging of single zebrafish islet slices, images were captured from the first quarter of the islet (depth of 10 to 15 μm) and imaged consistently at that depth for the duration of the time series. Fluorescence excitation was performed using a Chameleon two-photon laser (Coherent) tuned to 950 nm, and fluorescence emission was collected using a nondescanned binary GaAsP (BiG) detector with a custom-built filter cube containing an infrared light–blocked (500 to 550 nm) emission bandpass filter (Chroma) and a polarizing beamsplitter (Edmund Optics) to separate the parallel and perpendicular components of the emission.

### Image preprocessing and anisotropy calculation

Steady-state fluorescence anisotropy images were analyzed as previously described (*7*). Briefly, custom ImageJ and Python scripts were used to subtract the background, apply a threshold to the upper and lower fifth percentile of the range of intensity values, calculate the anisotropy value for every pixel including correcting for high NA skewing of the anisotropy, and report the average anisotropy of regions of interest (ROIs). Anisotropy (*r*) was calculated on a pixel-by-pixel basis from the parallel (*I*_∥_) and perpendicular (*I*_⊥_) components of the emission intensity as *r* = (*I*_∥_ − *GI*_⊥_)/(*I*_∥_ + 2*GI*_⊥_). *G* is the apparatus *G* factor, measured by imaging a fluorescein sample with and without a half-wave plate to correct for any biases in the detection system setup (*8*, *53*, *54*). Background subtraction and correction factors (*K*_a_, *K*_b_, and *K*_c_) were applied to the intensity values before anisotropy calculation. *K*_a_, *K*_b_, and *K*_c_ were calculated from the NA and index of refraction of the immersion medium and used to correct tilting of light by the high-NA lenses used (*8*, *54*, *55*). Analysis code is available at https://doi.org/10.5281/zenodo.8253493. Intensity and anisotropy images used for visual representation were background-subtracted and median-filtered before applying a lookup table.

### Quantification of anisotropy

Anisotropy of INS1E images was quantified by averaging the anisotropy of all cells in the same treatment group of an experimental replicate. For each individual cell, an ROI was drawn within the cell, and the anisotropy was calculated as the average anisotropy of all pixels in the ROI after preprocessing. Anisotropy of zebrafish slice images was quantified as the average anisotropy of all pixels in the slice at the specified time point or depth after preprocessing. Anisotropy of zebrafish whole-islet images was quantified as the average of all pixels in the preprocessed *z* stack, excluding slices containing less than 1000 pixels after preprocessing. Data points are plotted as the absolute anisotropy (“Anisotropy”) or as the change in anisotropy relative to a baseline (“∆Anisotropy”). For diamide-induced oxidation assays, separate curves of best fit were generated for the baseline (0 to 5 min), diamide oxidation (5 to 10 min), and diamide recovery (10 to 20 min) portions of each time series. The change in anisotropy over time was plotted as the difference in anisotropy relative to the 5-min time point. The diamide drop in anisotropy was quantified as the difference in anisotropy between the 10- and 5-min time points. The depletion half-life was quantified as the time required to reach half the maximum drop of the time series. The change in baseline anisotropy was quantified as the difference in anisotropy between the 20- and 5-min time points. The recovery half-life was quantified as the time required to reach half the maximum response for the diamide recovery portion of the time series.

### Statistical analysis

All data and graphical error bars are presented as means ± SEM. Statistical significance between the means of paired data was determined using a paired two-tailed *t* test in GraphPad Prism 6. Statistical significance between multiple groups was determined using one-way analysis of variance (ANOVA) followed by a post hoc test in GraphPad Prism 6. Dunnett’s multiple comparisons test was used to compare the mean of a control group to the means of multiple other groups. Sidak’s multiple comparisons test was used to compare the means of multiple groups.
