# Supplementary material for: Apollo-NADP+ reveals in vivo adaptation of NADPH/NADP+ metabolism in electrically activated pancreatic β cells
Source: Sci Adv. 2023 Oct 4;9(40):eadi8317. doi: 10.1126/sciadv.adi8317 (PMC10550227; doi:10.1126/sciadv.adi8317)
Supplement: Supplementary file 1 — Figs. S1 to S7 [file sciadv.adi8317_sm.pdf]

Science Advances
AAAS

Supplementary Materials for

Apollo-NADP$^+$ reveals in vivo adaptation of NADPH/NADP$^+$ metabolism in electrically activated pancreatic β cells

Cindy V. Bui *et al.*

Corresponding author: Jonathan V. Rocheleau, jon.rocheleau@utoronto.ca; Brian Ciruna, ciruna@sickkids.ca

Sci. Adv. **9**, eadi8317 (2023)
DOI: 10.1126/sciadv.adi8317

This PDF file includes:

Figs. S1 to S7

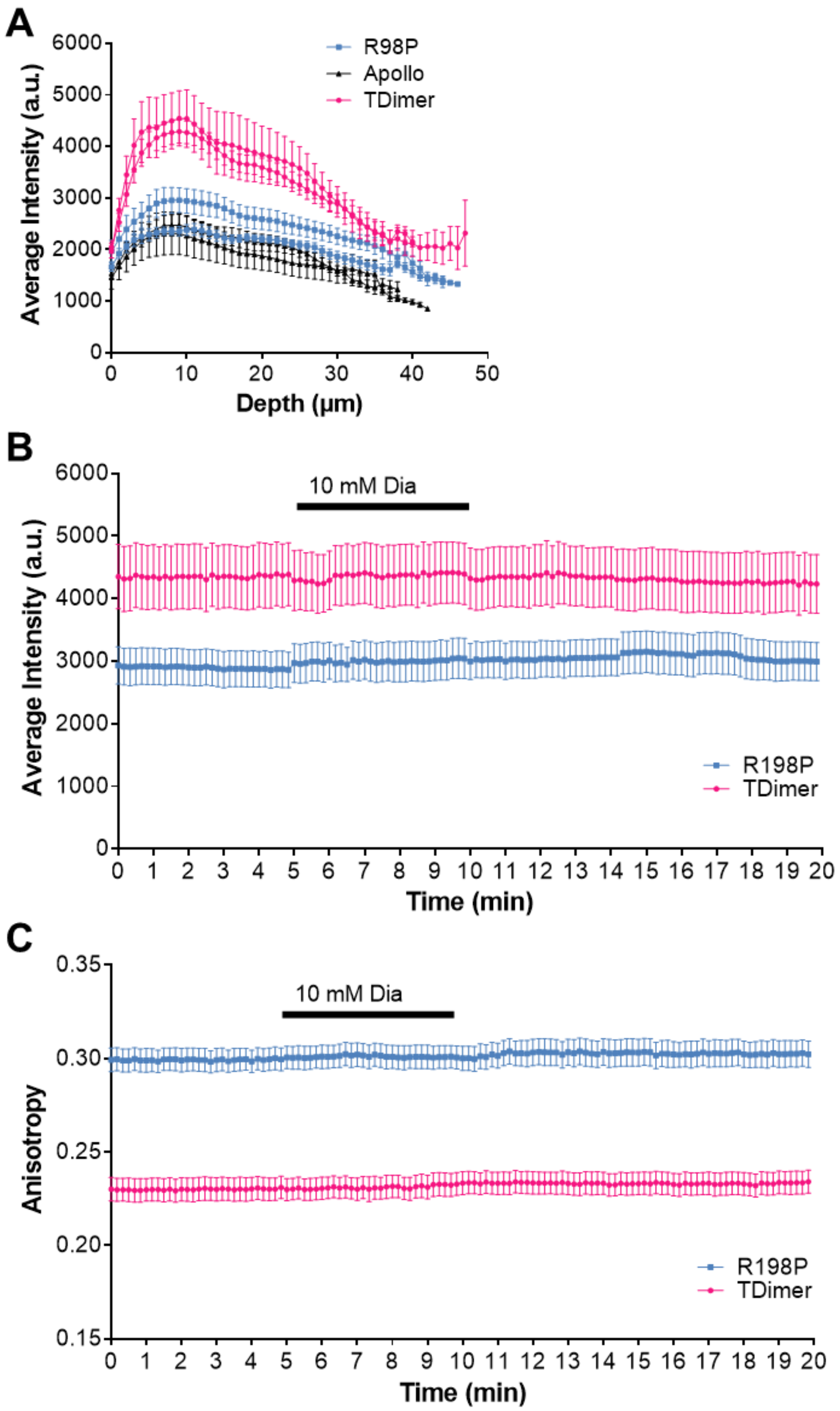

Fig. S1. ***In vivo*** **imaging of fluorescence anisotropy controls expressed in pancreatic β cells of 5 dpf zebrafish embryos.**

(**A**) *In vivo* fluorescence intensity imaging of pancreatic islets from 5 dpf zebrafish embryos, taken at 1 µm intervals. Each line represents embryo data from a distinct founder line. (**B**) *In vivo*

fluorescence intensity time series imaging of pancreatic islets from 5 dpf zebrafish embryos, taken at 10 sec intervals. An oxidizing agent, 10 mM diamide (Dia), was added at 5 min and removed at 10 min. (**C**) *In vivo* fluorescence anisotropy time series imaging of pancreatic islets from 5 dpf zebrafish embryos, taken at 10 sec intervals. 10 mM diamide was added at 5 min and removed at 10 min. n = 6-20 embryos.

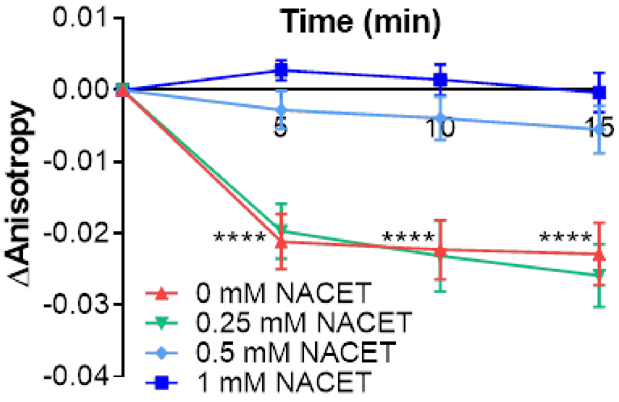

Fig. S2. Quantification of Apollo-NADP$^+$ diamide responses in live pancreatic β cells of 5 dpf zebrafish embryos treated with NACET.

In vivo time series fluorescence anisotropy imaging of 5 dpf islets of Apollo-NADP$^+$ transgenic zebrafish in response to treatment with 10 mM diamide, taken at 5 min intervals. Embryos were treated with the indicated concentration of NACET for 24 h prior to imaging. n = 5-11 embryos, ****$p < 0.0001$.

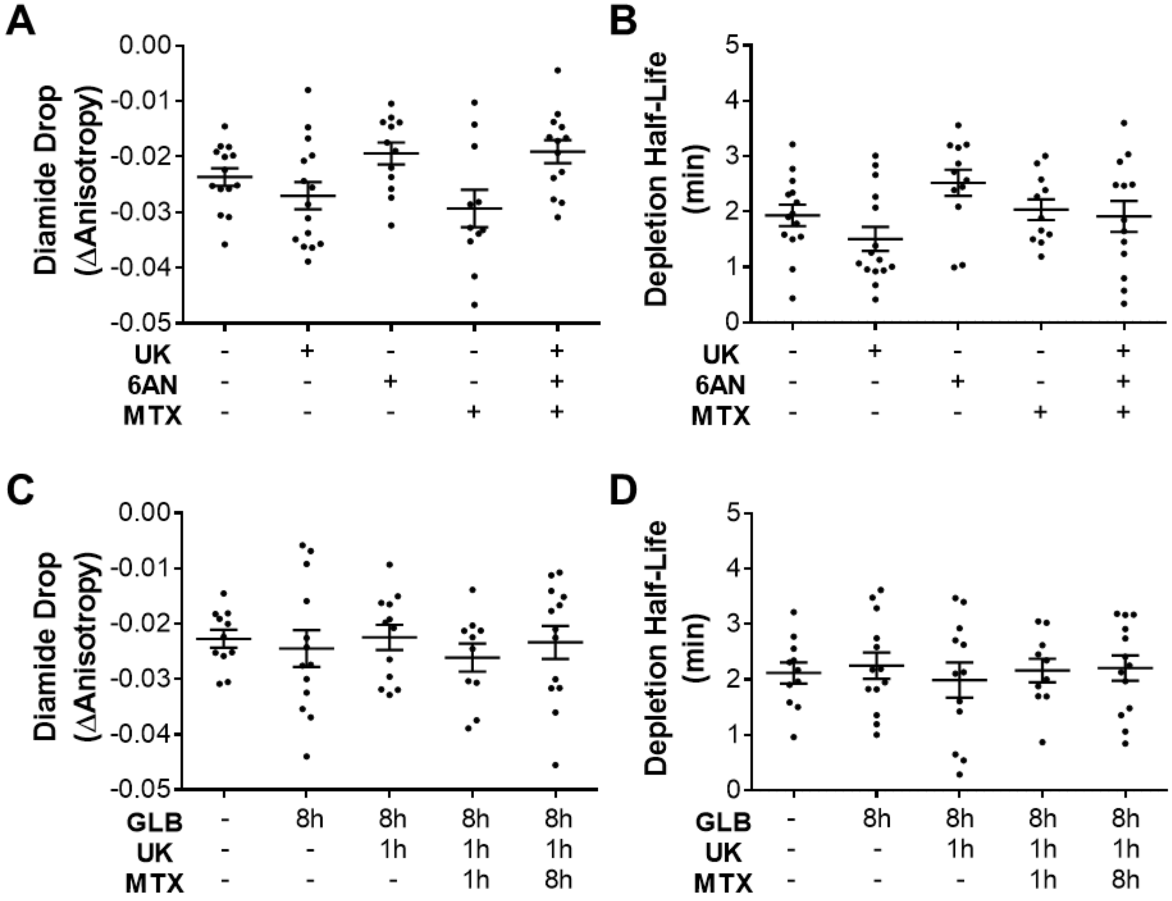

Fig. S3. Quantification of Apollo-NADP$^+$ responses to diamide treatment in live pancreatic β cells of 5 dpf zebrafish embryos.

(**A**) Change in anisotropy after 5 min treatment with 10 mM diamide, quantified from *in vivo* time series fluorescence anisotropy imaging of pancreatic islet slices from 5 dpf Apollo-NADP$^+$ transgenic zebrafish embryos. 200 µM UK was used to inhibit pyruvate cycling, 50 µM 6AN was used to inhibit pentose phosphate pathway, 50 µM MTX was used acutely to inhibit folate cycling (1 h) and chronically to also inhibit NADP$^+$ synthesis by NADK (8 h). Chemical inhibitors were added either 1 or 8 h prior to imaging as indicated. + indicates addition of an inhibitor 1 h prior to imaging. (**B**) NADPH depletion half-life in response to 10 mM diamide treatment, quantified from *in vivo* time series fluorescence anisotropy imaging of pancreatic islet slices from 5 dpf Apollo-NADP$^+$ transgenic zebrafish embryos. (**C**) Change in anisotropy after 5 min treatment with 10 mM diamide, quantified from *in vivo* time series fluorescence anisotropy imaging of pancreatic islet slices from 5 dpf Apollo-NADP$^+$ transgenic zebrafish embryos. Stress was induced by 8 h treatment with 20 µM GLB. (**D**) NADPH depletion half-life in response to 10 mM diamide treatment, quantified from *in vivo* time series fluorescence anisotropy imaging of pancreatic islet slices from 5 dpf Apollo-NADP$^+$ transgenic zebrafish embryos. n = 10-15 embryos.

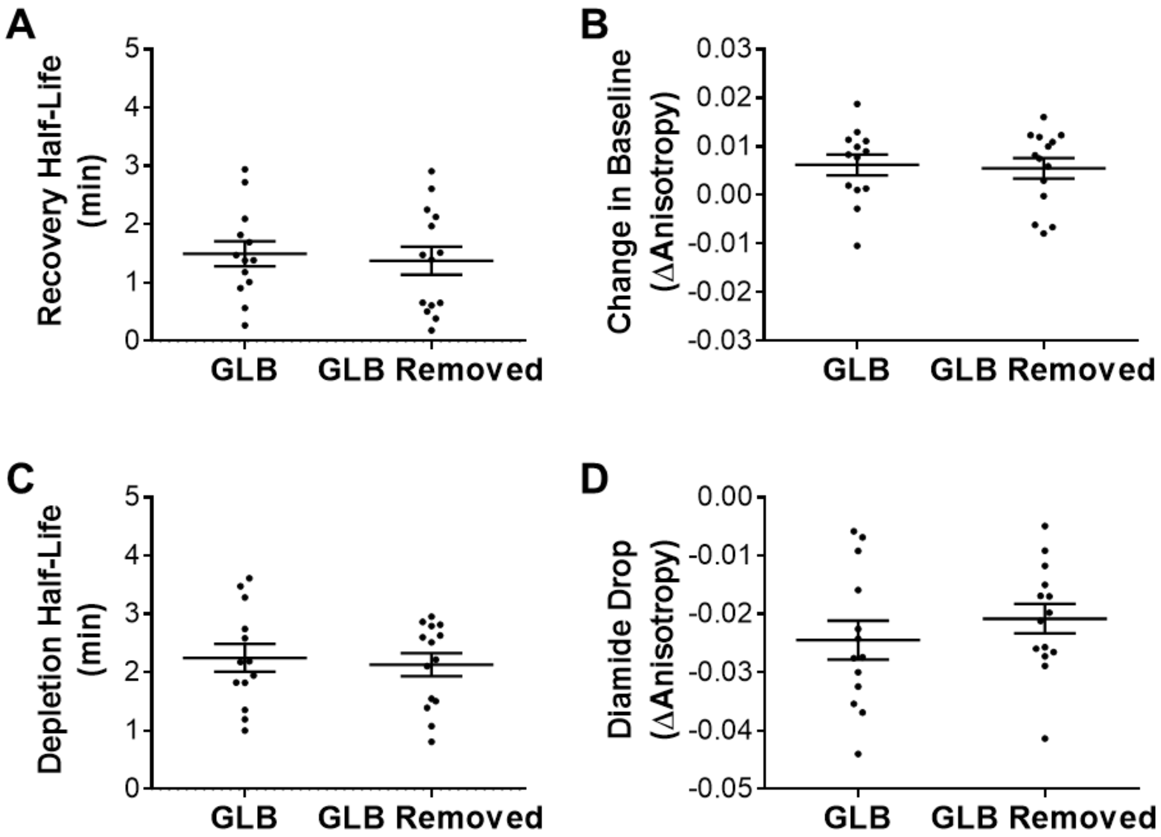

Fig. S4. Comparison of pancreatic β cell NADPH/NADP$^+$ dynamics between GLB present and removed during imaging of 5 dpf zebrafish embryos.

20µM GLB was present or removed after 8 h stress treatment. (**A**) NADPH recovery half-life after diamide removal, quantified from *in vivo* time series fluorescence anisotropy imaging of islet slices from 5 dpf Apollo-NADP$^+$ transgenic zebrafish embryos. (**B**) Change in baseline anisotropy, quantified from *in vivo* time series fluorescence anisotropy imaging of islet slices from 5 dpf Apollo-NADP$^+$ transgenic zebrafish embryos. (**C**) NADPH depletion half-life in response to 10 mM diamide treatment, quantified from *in vivo* time series fluorescence anisotropy imaging of pancreatic islet slices from 5 dpf Apollo-NADP$^+$ transgenic zebrafish embryos. (D) Change in anisotropy after 5 min treatment with 10 mM diamide, quantified from *in vivo* time series fluorescence anisotropy imaging of pancreatic islet slices from 5 dpf Apollo-NADP$^+$ transgenic zebrafish embryos. n = 13-14 embryos.

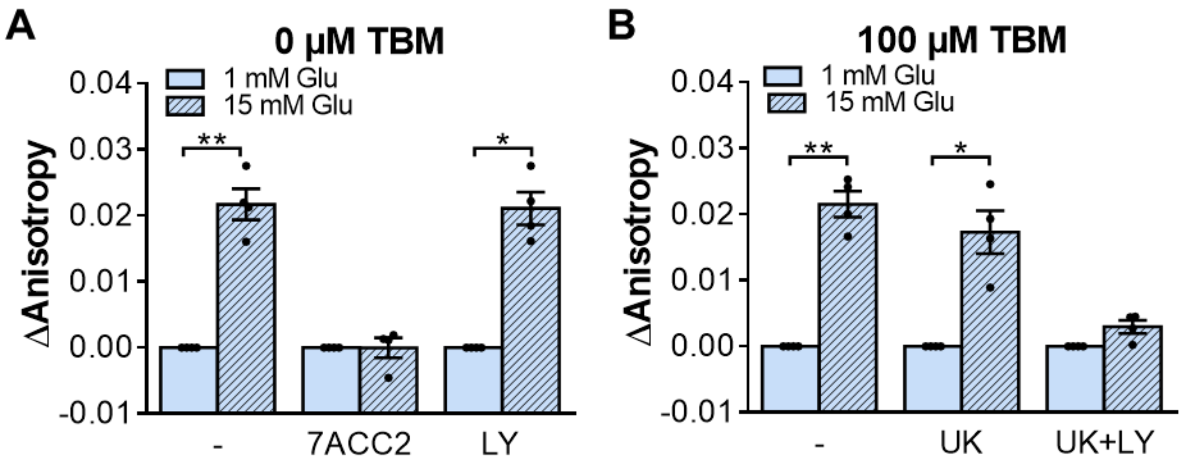

Fig. S5. Selective inhibition of NADP⁺ reduction pathways using alternative chemical inhibitors in unstressed and stressed INS1E β cells.

Glucose-stimulated $NADP^+$ reduction of INS1E β cells transfected with Apollo-$NADP^+$. (**A**) 10 µM 7ACC2 was used to inhibit pyruvate cycling and 10 µM LY was used to inhibit folate cycling. (**B**) Stress was induced for 8 h using 100 µM TBM. 50 µM UK was used to inhibit pyruvate cycling and 10 µM LY was used to inhibit folate cycling. Chemical inhibitors were added 1 h prior to imaging. n = 4 replicates, where $*p < 0.05$, $**p < 0.01$.

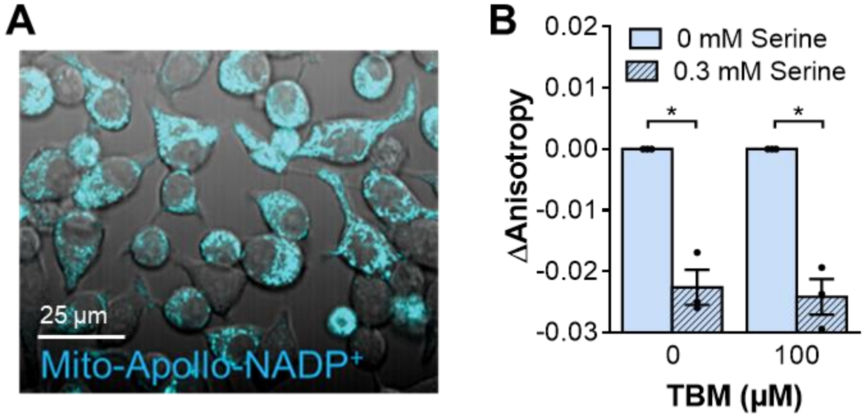

Fig. S6. Mitochondrial NADPH/NADP+ redox measurements of INS1E β cells in response to stress and serine stimulation.

(**A**) Fluorescence image of mitochondria-localized Apollo-NADP+ expressed in INS1E β cells. (**B**) 0.3 mM serine stimulation of unstressed (0 µM TBM) and stressed (8 h 100 µM TBM) INS1E β cells transfected with mitochondria-localized Apollo-NADP+. Cells were pre-treated with 1 mM asparagine for 1 h prior to imaging to promote extracellular serine uptake. Imaging was done at low glucose (1 mM) to avoid activation of pyruvate cycling. n = 3 replicates, $*p < 0.05$.

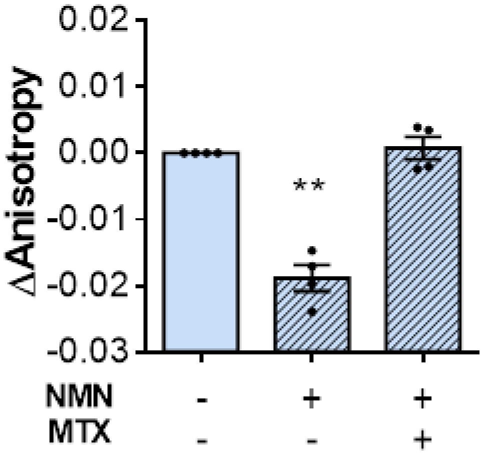

Fig. S7. Comparison of baseline anisotropy between INS1E pancreatic β cells treated with NMN alone or combined with MTX for 24 h.

INS1E β cells were transfected with Apollo-$NADP^+$ and treated 24 h prior to imaging at 1 mM glucose. Cells were treated with 100 μM NMN alone for 24 h to elevate NAD^+ levels or in combination with 5 μM MTX to block NAD^+ conversion to $NADP^+$ by NADK. n = 4 replicates, **$p < 0.01$.
